# The Antibiotic Era: Reform, Resistance, and the Pursuit of a Rational Therapeutics

**DOI:** 10.3201/eid2106.150212

**Published:** 2015-06

**Authors:** James A. Poupard

**Affiliations:** Center for the History of Microbiology/American Society for Microbiology Archives, Philadelphia, Pennsylvania, USA

**Keywords:** antibiotics, drugs, era, antimicrobial resistance, reform, drug resistance, rational therapeutics

There has been a flood of recent publications and media coverage on the current crisis relating to drug-resistant microorganisms. The Antibiotic Era: Reform, Resistance, and the Pursuit of a Rational Therapeutics takes a unique approach by presenting a well-documented history, starting in the earliest days with the introduction of antimicrobial drugs to the present time. It is built around those reformers who recognized problems relating to the irrational use of these agents and the difficulties these reformers experienced over many years.

Early chapters provide a firm foundation for the many issues that follow. They trace the development of the sulfa drugs and penicillin up to the period of World War II, and more important, the postwar introduction of the group of broad-spectrum “wonder-drug” antibiotics. The introduction of these drugs changed the practice of medicine but also stimulated the need for infectious disease experts to clarify their use and help counter the extreme and extensive marketing of these drugs by the pharmaceutical companies in the form of journal advertisements, supplements, and free physician samples.

As the author notes, experts like Maxwell Finland became “therapeutic rationalists” or “reformers” to help fill the vacuum left by the American Medical Association and the Food and Drug Administration because they only dealt with drug safety, not efficacy, and did not want to dictate therapeutic choices to practicing physicians. It was the introduction of the fixed-dose combination antibiotics in the 1950s, especially Panalba (tetracycline/novobiocin), that reinforced the need for real reform, a movement that dragged on for several decades before coming to fruition with the acceptance of the requirement for controlled clinical studies to prove the efficacy, as well as the safety, of antimicrobial agents.

Except for the last chapter, this well-researched book focuses exclusively on issues debated in the United States. This easy to read book is arranged in a logical chronologic order, and traces the complexity of newer antimicrobial drug classes and their related policy issues. The author provides 105 pages of references and documentation that makes this book a valuable source of information for historians of science, clinical microbiologists, and infectious disease physicians. If for no other reason, the extensive reference sections makes this book a wise investment for anyone researching or writing on these issues now or in the future, especially because many of the past issues are still being argued today.

